# Two New Tryptamine Derivatives, Leptoclinidamide and (-)-Leptoclinidamine B, from an Indonesian Ascidian *Leptoclinides dubius*

**DOI:** 10.3390/md10020349

**Published:** 2012-02-10

**Authors:** Hiroyuki Yamazaki, Defny S. Wewengkang, Teruaki Nishikawa, Henki Rotinsulu, Remy E. P. Mangindaan, Michio Namikoshi

**Affiliations:** 1 Department of Natural Product Chemistry, Tohoku Pharmaceutical University, Komatsushima, Aoba-ku, Sendai 981-8558, Japan; Email: yamazaki@tohoku-pharm.ac.jp (H.Y.); wdefny@yahoo.com (D.S.W.); rhenki@yahoo.com (H.R.); 2 Faculty of Fisheries and Marine Science, Sam Ratulangi University, Kampus Bahu, Manado 95115, Indonesia; Email: remysang@yahoo.com; 3 Laboratory of Taxonomy, Department of Biology, Faculty of Science, Toho University, Miyama, Funabashi 274-8510, Japan; Email: nishikawa@bio.sci.toho-u.ac.jp

**Keywords:** leptoclinidamide, leptoclinidamine, tryptamine, alkaloid, Indonesian ascidian, *Leptoclinides dubius*

## Abstract

Two new tryptamine-derived alkaloids, named as leptoclinidamide (**1**) and (-)-leptoclinidamine B (**2**), were isolated from an Indonesian ascidian *Leptoclinides dubius* together with *C*^2^-α-D-mannosylpyranosyl-L-tryptophan (**3**). The structure of **1** was assigned on the basis of spectroscopic data for **1** and its *N*-acetyl derivative (**4**). Compound **1** was an amide of tryptamine with two β-alanine units. Although the planar structure of **2** is identical to that of the known compound (+)-leptoclinidamine B (**5**), compound **2** was determined to be the enantiomer of **5** based on amino acid analysis using HPLC methods. Compounds **1** to **4** were evaluated for cytotoxicity against two human cancer cell lines, HCT-15 (colon) and Jurkat (T-cell lymphoma) cells, but none of the compounds showed activity.

## 1. Introduction

Ascidians are a rich source of biologically-active nitrogenous substances with high chemical diversity [1,2]. More than 80% of new compounds from ascidians contained nitrogen, and about 70% of nitrogenous compounds are alkaloids [3].

**Figure 1:**
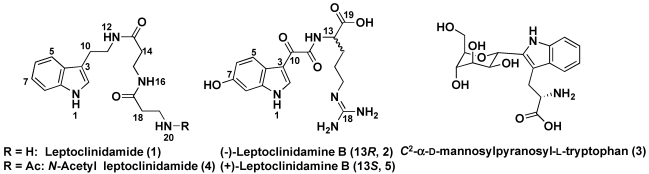
Structures of leptoclinidamide (**1**), (-)-leptoclinidamine B (**2**), and *C*^2^-α-D-mannosylpyranosyl-L-tryptophan (**3**).

In the course of our studies on the bioactive components from marine invertebrates, we found that the EtOH extract of an Indonesian ascidian *Leptoclinides dubius* inhibited the growth of *Escherichia coli*. Chemical study on the EtOH extract led to the isolation of two tryptamine-derived alkaloids, a *C*-glycosylated tryptophan and an antibacterial compound. Two alkaloids were revealed to be new compounds and named as leptoclinidamide (**1**) and (-)-leptoclinidamine B (**2**, Figure 1), and a tryptophan derivative was assigned as *C*^2^-α-D-mannosylpyranosyl-L-tryptophan (**3**) [4,5,6,7,8]. The major bioactive constituent could not be identified because the amount obtained from the ascidian was not enough to measure 2D NMR spectra. 

We report herein the isolation and structures of two new tryptamine-derived alkaloids leptoclinidamide (**1**) and (-)-leptoclinidamine B (**2**), which had unique amide moiety with two β-alanine units and D-arginine moiety, respectively.

## 2. Results and Discussion

The EtOH extract of an Indonesian ascidian *L. dubius* showed antimicrobial activity in the screening bioassay against *Escherichia coli* and was separated into nine fractions (fraction 1–fraction 9) by octadecylsilyl (ODS) column chromatography. Leptoclinidamide (**1**) was isolated from fraction 6 (50% MeOH eluate) by HPLC (ODS) and compounds **2** and **3** were obtained from fraction 3 (30% MeOH eluate) and fraction 2 (water eluate), respectively. The antibacterial activity against *E. coli* was detected in fraction 5 (50% MeOH eluate, 10 mm inhibition zone at 250 μg/disk), and HPLC separation of fraction 5 gave an antibacterial compound as a single HPLC peak.

Unfortunately, the antibacterial component did not give an informative ^1^H NMR spectrum because of the small amounts obtained, and, therefore, 2D NMR experiments could not be recorded. The structure of compound **3** was assigned on the basis of its spectral data and comparison with that of the reported values for *C*^2^-α-D-mannosylpyranosyl-L-tryptophan [6,7]. Compound **3** was first identified as a novel post-translationally modified tryptophan in human RNases [4] and isolated thereafter from the ascidian *L. dubius* in 2000 [8], but the role of compound **3** have not yet been elucidated.

Leptoclinidamide (**1**) was obtained as a TFA salt. The FAB-MS spectrum of **1** showed a peak at *m*/*z* 303.1821, and 16 ^13^C signals were detected in the ^13^C NMR spectrum (DMSO-*d_6_*). However, the ^1^H signals due to amine moiety were observed as broad peak in the NMR spectrum of **1**. Therefore, the molecular weight and formula of **1** were confirmed by the spectroscopic data for an *N*-acetyl derivative (**4**) of **1**. Compound **4** gave an [M + H]^+^ ion at *m*/*z* 345.1930 in the HRFAB-MS, and the molecular formula of **4** was determined as C_18_H_25_N_4_O_3_. Accordingly, the molecular formula of **1**, which revealed an [M + H]^+^ ion at *m*/*z* 303.1821 in the HRFAB-MS, were deduced as C_16_H_22_N_4_O_2_ (8 degrees of unsaturation).

**Figure 2:**
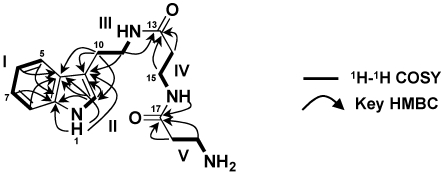
^1^H-^1^H COSY and HMBC correlations of leptoclinidamide (**1**).

The IR spectrum of **1** showed absorption bands at 1681 and 1584 cm^−1^, which were ascribable to two amide carbonyl groups. The ^13^C signals of **1** were classified into six methylene, four *sp*^2^ methine, one nitrogenated *sp*^2^ methine, two *sp*^2^ quaternary, one nitrogenated *sp*^2^ quaternary and two amide carbonyl carbons by the analysis of ^13^C NMR, DEPT and HMQC spectra of **1**. The ^1^H NMR spectrum (DMSO-*d_6_*) of **1** displayed 22 signals including *N*-H protons due to a primary amine (δ_H_ 7.66, 2H), two amides (δ_H_ 7.93 and 8.07), and an indole ring (δ_H_ 10.8). Table 1 shows ^1^H and ^13^C NMR data for **1** assigned by the analysis of ^1^H-^1^H COSY, HMQC and HMBC spectra. The ^1^H-^1^H COSY spectrum of **1** revealed the partial structures I through V (Figure 2). The presence of an indole ring was suggested by the ^1^H NMR signals at δ 7.09 (H-2), 7.48 (H-5), 6.93 (H-6), 7.02 (H-7), 7.29 (H-8), and 10.8 (1-NH) and HMBC correlations from these signals to the expected ^13^C NMR signals. HMBC correlations from H_2_-10 (δ_H_ 2.77) to C-2 (δ_C_ 122.5), C-3 (δ_C_ 111.8), and C-4 (δ_C_ 127.2) and from H_2_-11 (δ_H_ 3.29) to C-3 revealed a tryptamine unit in the molecule of **1**. The ^1^H NMR signals at δ 3.29 (H_2_-11) showed an HMBC correlation to one of two amide carbonyl carbons at δ_C_ 170.0 (C-13). The other amide linkage was determined by HMBC correlations to C-17 (δ_C_ 169.2) from 16-NH (δ_H_ 8.07), H_2_-18 (δ_H_ 2.38), and H_2_-19 (δ_H_ 2.93). These data suggested the structure of **1** as shown in Figure 1. The structure of **1** was further confirmed by the analysis of the *N*-acetyl derivative (**4**). The ^1^H NMR spectrum of **4** showed new signals corresponding to a methyl proton signal at δ 1.75 and a new amide proton signal at δ_H_7.78 and a corresponding loss of two primary amine proton signals detected in the ^1^H NMR spectrum of **1** (δ_H_7.66). These data led to the conclusion that leptoclinidamide has the structure **1** as shown in Figure 1.

**Table 1:** ^13^C (100 MHz) and ^1^H (400 MHz) NMR data for leptoclinidamide (**1**) in DMSO-*d_6_*.

No.	δ_C_	δ_H_ ( *J* in Hz)	HMBC
1-NH	-	10.8 brs	3, 4, 9
2	122.5	7.09 d (2.0)	3, 4, 9
3	111.8	-	
4	127.2	-	
5	118.1	7.48 d (8.0)	9
6	118.1	6.93 t (8.0)	4
7	120.8	7.02 t (8.0)	9
8	111.3	7.29 d (8.0)	4
9	136.2	-	
10	25.1	2.77 brt (7.4)	2, 3, 4
11	39.2	3.29 brt (7.4)	3, 13
12-NH	-	7.93 brt (5.8)	
13	170.0	-	
14	35.2	2.24 brt (7.4)	13
15	35.3	3.23 brt (7.4)	13
16-NH	-	8.07 brt (5.8)	17
17	169.2	-	
18	31.9	2.38 brt (7.4)	17
19	35.3	2.93 brt (7.4)	17
20-NH_2_	-	7.66 brs	

Compound **2** showed a molecular ion peak at *m/z* 362 [M + H]^+^ in the FAB-MS, and the molecular formula C_16_H_20_N_5_O_5_ was deduced from HRFAB-MS. The NMR spectrum of **2** revealed the presence of a disubstituted indole ring and an arginine moiety. A literature to search suggested the structure of (+)-leptoclinidamine B (**5**) as a candidate, and this structure was confirmed by comparing ^1^H and ^13^C NMR data for **2** with those for the reported values [9]. Compound **5** was originally isolated from an ascidian *Leptoclinides durus* and showed no apparent antiprotozoal or cytotoxic activity [9]. Although ^1^H and ^13^C NMR spectra of **2** were identical to those of **5**, the sign of the specific rotation of **2** was negative ([α]_D_ −20.6 in MeOH), suggesting that compound **2** was the enantiomer of **5** ([α]_D_ +27.0 in MeOH) [4]. To determine the absolute configulation of **2**, amino acid analysis was carried out by the methods described in the **Experimental Section**. The acid hydrolysate of **2** was analyzed by using chiral HPLC. In the analysis, an amino acid in the hydrolysate of **2** was detected at 3.7 min, which corresponded to D-Arg (L-Arg eluted at 11.3 min). This result was confirmed by Marfey’s method [10,11]. The hydrolysate of **2** was treated with Marfey’s reagent, 5-fluoro-2,4-dinitrophenyl-L-leucine amide (L-FDLA), and the resulting derivative was analyzed by HPLC using an ODS column. An L-FDLA derivative of an amino acid in the hydrolysate of **2** showed the same retention time as that of the derivative from D-Arg (13.4 min), whereas the L-FDLA derivative of L-Arg eluted at 15.9 min. Consequently, the absolute stereochemistry of **2** was determined to be 13*R*, as shown in Figure 1. Very recently, herdmanines A-D containing a D-Arg unit were isolated from a solitary ascidian *Herdmania momus* [12].

No biological activities have been reported for compound **3**. Compounds **1**–**4** were tested for their cytotoxicity against two human cancer cell lines (colon adenocarcinoma HCT-15 and T-cell leukemia Jurkat cells). However, none of the four compounds displayed activity against these cell lines at 30 μM. Since fractions 2, 3, and 6, from which respective compounds **3**, **2**, and **1** were isolated, did not show growth inhibitory activity against four microorganisms (see **Experimental Section**), compounds **1**–**3** will not have antimicrobial activity. Further study on biological activity of **1** is now in progress. More *L. dubius* will be collected from the same site to provide sufficient amounts of the antibacterial component for structural characterization.

## 3. Experimental Section

### 3.1. General

FAB-MS spectra were obtained using a JEOL JMS-MS 700 mass spectrometer (Tokyo, Japan). ^1^H and ^13^C NMR spectra were recorded on a JEOL JNM-AL-400 NMR spectrometer (400 MHz for ^1^H and 100 MHz for ^13^C) in DMSO-*d_6_* (δ_H_ 2.46, δ_C_ 39.5) or CD_3_OD (δ_H_ 3.31, δ_C_ 49.0). Optical rotations were measured with a JASCO P-2300 digital polarimeter (Tokyo, Japan). UV spectra were recorded on a Hitachi U-3310 UV-Visible spectrophotometer (Tokyo, Japan) and IR spectra on a PerkinElmer Spectrum One Fourier transform infrared spectrometer (Waltham, MA, USA). Preparative HPLC was carried out with a Hitachi L-6200 system.

### 3.2. Materials

Fetal bovine serum (FBS) and other culture materials were purchased from Invitrogen (Carlsbad, CA, USA). 3-(4,5-Dimethylthiazol-2-yl)-2,5-diphenyltetrazolium bromide (MTT) was purchased from Sigma-Aldrich (St. Louis, MO, USA). All other chemicals and organic solvents were purchased from Wako Pure Chemical Industries Ltd. (Osaka, Japan).

### 3.3. Ascidian

The ascidian was collected by scuba diving at the coral reef in the Lembeh Strait, North Sulawesi, Indonesia in October 2009 and identified as *Leptoclinides dubius* by T. N. The voucher specimen is deposited at the National Museum of Nature and Science, Tokyo as NSMT Pc-1123.

### 3.4. Extraction and Isolation

The ascidian (250 g, wet weight) was cut into small pieces and soaked in EtOH on a boat immediately after collection. The organism was further extracted twice with EtOH. The EtOH extract (3.57 g) was suspended in H_2_O and adsorbed on an ODS column (100 g). The ODS column was eluted stepwise with 0, 30, 50, 70, and 100% MeOH in 0.10% TFA aqueous solution into nine fractions (fraction 1–fraction 9). Fraction 2, eluted with H_2_O, was concentrated to yield a red brown oil (108.0 mg), and 20.0 mg of the fraction was purified by preparative HPLC column, PEGASIL ODS (10 mm × 250 mm); solvent, 35% MeOH containing 0.10% TFA; flow rate, 2.0 mL/min; detection, UV at 210 nm] to give compound **3** (eluted at 9.9 min) as a colorless solid (6.0 mg). Fraction 3, eluted with 30% MeOH, was concentrated to yield a red brown oil (399.5 mg), and 20.0 mg was fractionated by preparative HPLC (same conditions as Fraction 2) to yield compound **2** (eluted at 11.0 min) as a pale yellow oil (4.0 mg). Fraction 5 (95. 0 mg), eluted with 50% MeOH, was active against *E. coli* and subjected to ODS HPLC with 40% MeOH containing 0.10% TFA (the other conditions were the same as above) into six fractions (Fraction 5-1–Fraction 5-6). Fraction 5-6 showed the growth inhibition against *E. coli*, but the amounts were not enough to obtain a good ^1^H NMR spectrum. Fraction 6, eluted with 50% MeOH, was concentrated to dryness (50.5 mg), and 20.0 mg was separated by preparative HPLC with the same conditions as Fraction 5 and afforded compound **1** (eluted at 15.0 min) as a colorless oil (2.0 mg).

**Leptoclinidamide (1)**: obtained as TFA salt; UV λ_max_ (MeOH) nm (log ε): 202 (4.47), 221 (4.31); IR ν_max_ (KBr) cm^−1^: 3418, 2938, 1680, 1584, 1556, 1457; HRFAB-MS (*m/z*) found: 303.1821, calcd: 303.1821 [M + H]^+^ for C_16_H_23_N_4_O_2_; ^1^H and ^13^C NMR data, see Table 1.

**(-)-Leptoclinidamine B (2)**: obtained as TFA salt; [α]^20^_D_ −20.6 (*c* = 0.033, MeOH); UV λ_max_ (MeOH) nm (log ε): 203 (4.38), 247 (3.78), 284 (3.78), 334 (3.22); IR ν_max_ (KBr) cm^−1^: 3384, 1684, 1638, 1450, 1275, 1131; HRFAB-MS (*m/z*) found: 362.1465, calcd: 362.1464 [M + H]^+^ for C_16_H_20_N_5_O_5_; ^1^H-NMR (DMSO-*d_6_*) δ_H_ 1.53 (2H, m, H-15), 1.77, 1.86 (2H, m, H-14), 3.14 (2H, m, H-16), 4.28 (1H, brs, H-13), 6.75 (1H, d, *J* = 8.0 Hz, H-6), 6.87 (1H, s, H-8), 7.12 (4H, brs, 18-N_2_H_4_), 7.62 (1H, brs, 17-NH), 7.97 (1H, d, *J* = 8.0 Hz, H-5), 8.52 (1H, s, 12-NH), 8.72 (1H, brd, H-2), 9.33 (1H, brs, 7-OH), 11.9 (1H, brs, 1-NH); ^13^C-NMR (DMSO-*d_6_*) δ_C_25.2 (C-15), 27.9 (C-14), 40.3 (C-16), 51.9 (C-13), 97.2 (C-8), 112.4 (C-6), 112.5 (C-3), 118.8 (C-4), 121.7 (C-5), 137.3 (C-2), 137.5 (C-9), 154.6 (C-7), 156.7 (C-18), 163.5 (C-11), 173.0 (C-20), 181.3 (C-10).

***C*^2^-α-D-mannosylpyranosyl-L-tryptophan (3)**: obtained as TFA salt; [α]^20^_D_ +15.5 (*c* = 0.52, MeOH); FAB-MS (*m/z*): 367 [M + H]^+^; ^1^H-NMR (CD_3_OD) δ_H_ 3.31, 3.65 (2H, m), 3.65 (1H, m), 3.65, 4.29 (2H, m), 3.89 (1H, m), 3.97 (1H, m), 4.05 (1H, brd), 4.25 (1H, brdd), 5.04 (1H, brd), 7.07 (1H, t, *J* = 7.8 Hz), 7.11 (1H, t, *J* = 7.8 Hz), 7.38 (1H, d, *J* = 7.8 Hz), 7.63 (1H, d, *J* = 7.8 Hz).

### 3.5. Synthesis of N-Acetyl Derivative *(**4**)* of Compound ***1***

Acetic anhydride (120 μL) was added to a solution of fraction 6 (20 mg) in MeOH (340 μL) at room temperature. The mixture was stirred for 12 h and evaporated. The residue was purified by preparative HPLC (40% MeOH containing 0.10% TFA) using ODS column (PEGASIL ODS) to give 1.0 mg of **4**: HRFAB-MS (*m/z*) found: 345.1930, calcd: 345.1927 [M + H]^+^ for C_18_H_25_N_4_O_3_; ^1^H-NMR (DMSO-*d_6_*) δ_H_ 1.75 (3H, s), 2.17 (2H, t, *J* = 7.6 Hz), 2.21 (2H, t, *J* = 7.6 Hz), 2.79 (2H, t, *J* = 7.6 Hz), 3.18 (2H, t, *J* = 7.6 Hz), 3.22 (2H, t, *J* = 7.6 Hz), 3.31 (2H, t, *J* = 7.6 Hz), 6.95 (1H, t, *J* = 7.4 Hz), 7.04 (1H, t, *J* = 7.4 Hz), 7.11 (1H, d, *J* = 8.3 Hz), 7.31 (1H, d, *J* = 8.3 Hz), 7.50 (1H, d, *J* = 2.0 Hz), 7.78 (NH, 1H, brt, *J* = 5.4 Hz), 7.85 (NH, 1H, brt, *J* = 5.8 Hz), 7.93 (NH, 1H, brt, *J* = 5.8 Hz), 10.8 (NH, 1H, brs).

### 3.6. Acid Hydrolysis of Compound ***2***

Compound **2** (0.5 mg) was suspended in 6.0 *N* HCl (1.0 mL) and heated at 100 °C for 8 h. The mixture was cooled to room temperature and evaporated to dryness.

### 3.7. Chiral HPLC of Peptide Hydrolysate

Hydrolysate of **2** was dissolved in H_2_O (0.4 mL) and 5.0 μL was analyzed by HPLC using the following condition (A): L-6200 system; column, SUMICHIRAL OA-6000 (Sumika Chemical Analysis Service, Ltd., Tokyo, Japan), 4.6 × 150 mm; flow rate, 1.0 mL/min; detection, UV 254 nm; mobile phase, 2 mM CuSO_4_ in 2.0% CH_3_CN. Authentic L- and D-Arg were eluted at 11.3 and 3.7 min, respectively. An amino acid in the hydrolysate of **2** was detected at 3.7 min.

### 3.8.Marfey’s Analysis

Hydrolysate of **2** was dissolved in 1 M NaHCO_3_ (200 μL) and reacted with 1% L-FDLA (100 μL in acetone) at 40 °C for 2 h. After cooling, the sample was quenched with 1 *N* HCl and dried under vacuum. The solid residue was dissolved in 50% aq CH_3_CN (400 μL) and analyzed under the following condition (B): L-6200 system; column, Pegasil ODS SP100 (Senshu Scientific), 4.6 × 250 mm; flow rate, 0.8 mL/min; detection, UV 340 nm; mobile phase, a liner gradient from 30% to 60% CH_3_CN containing 0.05% TFA. L-FDLA derivatives of authentic L- and D-Arg eluted at 15.9 and 13.4 min, respectively, and the L-FDLA derivative in the hydrolysate of **2** was detected at 13.4 min.

### 3.9. Antimicrobial Assay

The growth inhibitory activity was examined by the paper disk method against *Mucor hiemalis* IAM 6088 (fungus), *Saccharomyces cerevisiae* IAM 1438T (yeast), *Staphylococcus aureus* IAM 12544T (Gram-positive bacterium), and *Escherichia coli* IAM 12119T (Gram-negative bacterium) as test microorganisms. A paper disk containing 250 μg of the test sample was placed on a test plate.

### 3.10. Cytotoxicity Assay

HCT-15 and Jurkat cells were obtained from the Center for Biomedical Research, Institute of Development, Aging, and Cancer, Tohoku University (Miyagi, Japan). Two cell lines were cultured in RPMI-1640 medium. The medium was supplemented with 10% fetal bovine serum, 100 units/mL penicillin, and 100 μg/mL streptomycin. Exponentially growing cells cultured in a humidified chamber at 37 °C containing 5.0% CO_2_ were used for the experiments.

Cytotoxic activity was evaluated using the colorimetric MTT assay [13]. HCT-15 cells (1.0 × 10^4^ cells in 100 μL) or Jurkat cells (2.0 × 10^4^ cells in 100 μL) were added to each well of a 96-well plastic plate (Corning Inc., Corning, NY, USA). A sample (1.0 μL in MeOH) was added to each well to make the final concentration from 0 to 27 μM and the cells were incubated for 48 hours at 37 °C. MTT (10 μL of 5.5 mg/mL stock solution) and a cell lysate solution (90 μL, 40% *N,N*-dimethylformamide, 20% sodium dodecyl sulfate, 2.0% CH_3_COOH and 0.030% HCl) were added to each well, and the plate was shaken thoroughly by agitation at room temperature for overnight. The optical density of each well was measured at 570 nm using an MTP-500 microplate reader (Corona Electric Co., LTD., Ibaraki, Japan).

## 4. Conclusions

Two new tryptamine derivatives, leptoclinidamide (**1**) and (-)-leptoclinidamine B (**2**), were isolated from the EtOH extract of an Indonesian ascidian *Leptoclinides dubius* together with a known compound, *C*^2^-α-D-mannosylpyranosyl-L-tryptophan (**3**). Biological activity of compound **3** has not been clarified, and, in this study, we found that compounds **1**–**4** were not active against two human cancer cell lines (HCT-15 and Jurkat) and four microorganisms (Gram positive and negative bacteria, yeast, and fungus). An antibacterial component against *E. coli* was also obtained from the EtOH extract, but the structure has not been determined because the amounts were not enough to measure 2D NMR spectra.

## References

[1] Faulkner D.J. (2002). Marine natural products. Nat. Prod. Rep..

[2] Blunt J.W., Copp B.R., Munro M.H.G., Northcote P.T., Prinsep M.R. (2011). Marine natural products. Nat. Prod. Rep..

[3] Wang W.F., Namikoshi M. (2007). Bioactive nitrogenous metabolites from ascidians. Heterocycles.

[4] De Beer T., Vliegenthart J.F.G., Loffler A., Hofsteenge J. (1995). The hexopyranosyl residue that is *C*-glycosidically linked to the side chain of tryptophan-7 in human RNase Us is α-mannopyranose. Biochemistry.

[5] Gutsche B., Grun C., Scheutzow D., Herderich M. (1999). Tryptophan glycoconjugates in food and human urine. Biochem. J..

[6] Manabe S., Ito Y. (1999). Total synthesis of novel subclass of glyco-amino acid structure motif: *C^2^*-α-L-*C*-mannosylpyranosyl-L-tryptophan. J. Am. Chem. Soc..

[7] Nishikawa T., Ishikawa M., Isobe M. (1999). Synthesis of a α-*C*-mannosyltryptophan derivative, naturally occurring *C*-glycosyl amino acid found in human ribonuclease. Synlett.

[8] Garcia A., Lenis L.A., Jimenez C., Debitus C., Quinoa E., Riguera R. (2000). The occurrence of the human glycoconjugate *C^2^*-α-D-mannosylpyranosyl-L-tryptophan in marine ascidians. Org. Lett..

[9] Carroll A.R., Avery V.M. (2009). Leptoclinidamines A-C, indole alkaloids from the Australian ascidian *Leptoclinides durus*. J. Nat. Prod..

[10] Marfey P. (1984). Determination of D-amino acids. II. Use of a bifunctional reagent, 1,5-difluoro-2,4-dinitrobenzene. Carlsberg Res. Commun..

[11] Kuo H.K., Kai K., Akiyama K., Hayashi H. (2012). Novel bioactive peptides, PF1171F and PF1171G, from unidentified ascomycete OK-128. Tetrahedron Lett..

[12] Li J.L., Han S.C., Yoo E.S., Shin S., Hong J., Cui Z., Li H., Jung J.H. (2011). Anti-inflammatory amino acid derivatives from the ascidian *Herdmania momus*. J. Nat. Prod..

[13] Mosmann T. (1983). Rapid colorimetric assay for cellular growth and survival: application to proliferation and cytotoxicity assays. J. Immunol. Methods.

